# Evolutionary Dynamics Analysis of Human Metapneumovirus Subtype A2: Genetic Evidence for Its Dominant Epidemic

**DOI:** 10.1371/journal.pone.0034544

**Published:** 2012-03-30

**Authors:** Jianguo Li, Lili Ren, Li Guo, Zichun Xiang, Gláucia Paranhos-Baccalà, Guy Vernet, Jianwei Wang

**Affiliations:** 1 MOH Key Laboratory of Systems Biology of Pathogens and Christophe Mérieux Laboratory, IPB, CAMS-Fondation Mérieux, Institute of Pathogen Biology (IPB), Peking Union Medical College (PUMC) & Chinese Academy of Medical Sciences (CAMS), Beijing, People's Republic of China; 2 Fondation Mérieux, Lyon, France; University of Illinois at Chicago, United States of America

## Abstract

Human metapneumovirus (hMPV) is a respiratory viral pathogen in children worldwide. hMPV is divided into four subtypes: hMPV_A1, hMPV_A2, hMPV_B1, and hMPV_B2. hMPV_A2 can be further divided into hMPV_A2a and A2b based on phylogenetic analysis. The typical prevalence pattern of hMPV involves a shift of the predominant subtype within one or two years. However, hMPV_A2, in particular hMPV_A2b, has circulated worldwide with a several years long term high epidemic. To study this distinct epidemic behavior of hMPV_A2, we analyzed 294 sequences of partial G genes of the virus from different countries. Molecular evolutionary data indicates that hMPV_A2 evolved toward heterogeneity faster than the other subtypes. Specifically, a Bayesian skyline plot analysis revealed that hMPV_A2 has undergone a generally upward fluctuation since 1997, whereas the other subtypes experienced only one upward fluctuation. Although hMPV_A2 showed a lower value of mean dN/dS than the other subtypes, it had the largest number of positive selection sites. Meanwhile, various styles of mutation were observed in the mutation hotspots of hMPV_A2b. Bayesian phylogeography analysis also revealed two fusions of diffusion routes of hMPV_A2b in India (June 2006) and Beijing, China (June 2008). Sequences of hMPV_A2b retrieved from GenBank boosted simultaneously with the two fusions respectively, indicating that fusion of genetic transmission routes from different regions improved survival of hMPV_A2. Epidemic and evolutionary dynamics of hMPV_A2b were similar to those of hMPV_A2. Overall, our findings provide important molecular insights into hMPV epidemics and viral variation, and explain the occurrence of an atypical epidemic of hMPV_A2, particularly hMPV_A2b.

## Introduction

Human metapneumovirus (hMPV), first identified in 2001 in the Netherlands, is an enveloped, non-segmented, RNA virus that belongs to the *Paramyxoviridae* family and *pneumovirus* subfamily [1]. It is an important respiratory viral pathogen in children, which circulates in all age groups worldwide, causing illnesses that range from mild colds to severe lower respiratory tract diseases [1]–[3].

The genome of hMPV is approximately 13 kb in length and is comprised of eight genes, encoding nucleocapsid (N), phosphoprotein (P), matrix protein (M), fusion glycoprotein (F), transcriptional enhancer protein (M2-1), small hydrophobic protein (SH), attachment glycoprotein (G), and a large polymerase protein (L) [4]. As a type II membrane protein, the hMPV G protein plays an important role in viral attachment and is a potential target for neutralizing and protective immune responses [5], [6]. Accordingly, the G protein shows extensive amino acid variations, mostly in the ectodomain, that help the virus to escape immunity pressure from hosts [7], [8]. Thus, the G protein has been widely used to infer evolutionary relationships among isolates from different regions [8]–[10]. Based on genetic variability of the G gene, hMPV has been divided into two genotypes (A and B) and four subtypes (A1, A2, B1, and B2) [8], [11]. There are two subgroups of hMPV_A2, hMPV_A2a and hMPV_A2b, the latter of which was identified in Germany in 2006 [12].

The prevalence pattern of hMPV is similar to that of respiratory syncytial virus; two subgroups of the virus can co-circulate with varying degrees of prevalence [13], [14]. Shifts in the predominant hMPV subtype within one or two years are typical in the prevalence pattern of hMPV, and can be attributed to the response to the existing immunity against circulating strains [11]–[12], [15]–[19]. However, such a shift has not been observed in the prevalence pattern of the hMPV_A2 subtype. Reports from Korea, Mexico, Italy, and Germany indicate that hMPV_A2 can maintain predominance for three or more years, rather than undergoing the shift observed in other hMPV subtypes [15], [20]–[23]. The mechanism behind this distinctive epidemic feature of hMPV_A2 is unclear.

Here, we compiled the epidemic patterns of the hMPV subtypes based on the global incidences of hMPV subtypes, using published data as well as our own data collected between 1999 and 2009. We found that hMPV_A2 was predominant from 2002 to 2009. To interpret the mechanisms behind this phenomenon, we reconstructed and compared the evolutionary dynamics of all hMPV subtypes, particularly the phylodynamics of hMPV_A2a and A2b, based on sequences of the G gene. Our findings provide insights into the prevalence pattern of the hMPV subtypes and the evolutionary mechanism behind the atypical prevalence pattern of hMPV_A2.

## Materials and Methods

### Sample collection

A total of 60 hMPV-positive respiratory specimens were taken from pediatric and adult patients with acute respiratory tract infections (ARTIs) at the Beijing Children Hospital and the Peking Union Medical College Hospital in Beijing, China between 2006 and 2009. Sampling was performed once for each patient. The inclusion criteria of ARTI patients have been described previously [24], [25]. Briefly, the enrolled patients had acute respiratory symptoms, acute fever (body temperature ≧38.0°C), and normal or low leukocyte count. For each adult patient, a nasal and a throat swab were collected simultaneously. The two swabs were combined in one tube containing 3 ml viral transport medium (VTM). For each pediatric patient, about 3 ml of nasopharyngeal aspirate (NPA) was collected under aseptic conditions. All samples were stored at −80°C prior to analysis. Clinical information of each enrolled patient was recorded in a standard form and reviewed retrospectively upon identification of hMPV-positive patients. Written informed consent was obtained from all participants or guardians on behalf of the minors/children participants. The study was approved by the Medical Ethic Review Board of the Institute of Pathogen Biology, Chinese Academy of Medical Sciences. All samples were coded prior to analysis to ensure anonymity.

### Nucleotide amplification and sequencing of hMPV partial G genes

RNA was extracted from the samples using QIAamp viral RNA mini Kit (Qiagen, Duesseldorf, Germany), and reverse transcribed using SuperScript II reverse transcription system (Invitrogen, Carlsbad, CA) following the manufacturer's instructions. The cDNA of hMPV was amplified by nested PCR. The 20 µl PCR reaction mixture contained 2 µl of reaction mix, 0.4 µl of dNTP (10 µM), 0.2 µl of each primer (50 µM) and 0.2 µl of Taq Polymerase. Primers for hMPV genotype A were: outer sense 42-CAAAGCAAGAGTGAAAAATCGTGTG′, inner sense 44- AAGCAAGAGTGAAAAATCGTGTGGC, outer antisense 681- TGTTTTTACAATGTTGGTGTGCTAT, and inner antisense 527- GTGGTGGCTCTGGGGACCGCCTTCG. Primers for hMPV genotype B were: outer/inner sense 104-TTATTGGACTAACAGCGTTAAGCAT, outer antisense 805- ATGGTGTCTATGTTTTTCTGGGTCT, inner antisense 736- TTTTTGTTAACTACTTGGATGGGAT. Two microliters of cDNA was used as template in the first reaction, and 2 µl of the first round product was used as template in the second reaction. The two-round nested PCR was performed using these cycling conditions: pre-denaturing at 94°C for 180 s; 30 cycles of amplification at 94°C for 30 s; 50°C for 30 s; and 72°C for 60 s, with a terminal elongation step at 72°C for 10 min. The sequence data obtained for the hMPV G gene was deposited to GenBank under accession numbers JN166985 to JN167044 (Table S1).

### hMPV sequence collection and phylogenetic analysis

A total of 234 partial G gene cDNA sequences (www.ncbi.nlm.nih.gov), derived from samples with known background information including collection dates (between 1981 and 2009) and collection places, were obtained from GenBank. Those sequences plus the 60 sequences obtained in this study were subjected to phylogenetic analysis. These 294 sequences represent hMPV strains from 12 countries and cover most encoding sequences of the G protein's ectodomain. To avoid possible sampling bias, all the sequences used in this study were obtained from simultaneous screening of all four hMPV subtypes. The background information of the G gene sequences enrolled in this study is provided in Table S1.

Estimates of genetic distance were used to evaluate genetic divergence between hMPV subtypes [26]. The overall mean distances of the hMPV subtypes were estimated using MEGA (version 4.0) [27]. A kimura-2 parameter model, gamma-distributed among-site rate variation with four rate categories (γ4), and 1000 bootstraps were applied for distance estimation.

Phylogenetic reconstructions of the hMPV subtypes were performed by maximum-likelihood (ML) and neighbor-joining (NJ) methods using PAUP*(Phylogenetic Analysis Using Parsimony* and other methods, version 4b10 beta) [28], and by the Bayesian Markov Chain Monte Carlo (MCMC) method using BEAST (Bayesian evolutionary analysis by sampling trees version 1.5.4) [29]. The best fit nucleotide substitution model (s) for ML and the Bayesian MCMC analysis were selected using Modeltest (version 3.7) [30].

### Bayesian MCMC evolutionary analysis

Evolutionary parameters, including molecular clock phylogenies, demographic histories, evolution rates, and divergence times, were jointly estimated from heterochronous G genes of hMPV subtypes and hMPV_A2 sublineages using the Bayesian MCMC method implemented in BEAST (version 1.5.4). Bayesian MCMC analysis was performed using the uncorrelated lognormal-distributed model (UCLD). Analysis was performed using the best fit nucleotide substitution model general time reversible (GTR) with a gamma-distributed among-site rate variation with four rate categories (γ4) as estimated by Modeltest. To determine the degree to which dating estimates were affected by the demographic tree model, chosen Bayesian MCMC analyses were repeated using the constant size and exponential growth tree models. Each Bayesian MCMC analysis was run for 200 million states and sampled every 5,000 states. Posterior probabilities were calculated using Tracer (version 1.5) with a burn-in of 2 million states. Bayesian skyline plots for hMPV_A2a and A2b were estimated to depict the relative viral genetic diversity (*g*) over time.

### Natural selection and adaptation analysis

Selection pressures on the hMPV G genes were estimated from the ratios of nonsynonymous to synonymous substitution (dN/dS) using the codon-based phylogenetic method in CODEML (distributed in PAML, version 4) [31]. The M2a codon substitution model of CODEML can estimate three classes of sites: purifying (0≤ω<1), neutral (ω = 1), and positive selection (ω>1) sites. Posterior probabilities of the inferred positive selection sites were calculated using the Bayes empirical Bayes (BEB) approach which accounts for sampling errors. Only the sites with a posterior probability of ω>95% were considered as positive selection sites. Because most viral amino acids are encoded by more than one codon, a larger value of dN/dS does not always mean that the chance of an amino acid switch to any given position is higher. To infer the positive selection sites at the amino acid level, the deduced amino acid sequence entropy was determined using BioEdit (version 7.0.9) [32].

To chronologically trace the evolution of nonsynonymous changes throughout the evolutionary history of hMPV_A2, the nonsynonymous changes from the heterochronous virus sequences were reconstructed using a joint maximum likelihood method using HyPhy software version 0.99 [33]. The occurrence time of each nonsynonymous substitution in hMPV_A2 was estimated using the maximum clade credibility (MCC) tree generated from the Bayesian MCMC molecular clock analysis [29].

### Phylogeography analysis

A Bayesian stochastic search variable selection (BSSVS) approach, included in Beast 1.5.4, that allows the exchange rates in the CTMC to be zero with some prior probability, was used to find a minimal set of rates demonstrating the diffusions in the phylogeny [34]. To identify the rates contributing to the migration path, a Bayes factor test was performed by comparing the posterior to prior odds that individual rates are zero. BF>3 was considered significant.

A maximum clade credibility tree was applied to summarize the obtained trees using TreeAnnotator which is included in BEAST 1.5.4. To visualize the diffusion rates over time, the location-annotated MCC tree was converted to a keyhole markup language (KML), which is suitable for viewing with GoogleEarth (http://earth.google.com).

## Results

### Global epidemics of hMPV subtypes

**Figure 1:**
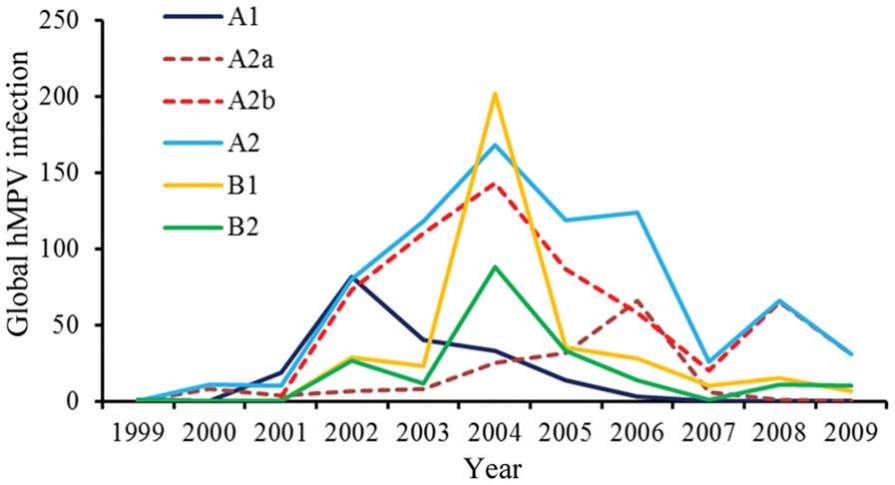
Global frequencies of hMPV subtypes and hMPV_A2 sublineages between 1999 and 2009. The numbers of global hMPV infection were compiled from published reports of hMPV prevalence by PubMed index (www.ncbi.nlm.nih.gov/pubmed). Only reports that screened all four subtypes of hMPV simultaneously were used. The numbers on Y-axis are yearly total qualified sequences of hMPV.

Epidemiological reports on hMPV subtypes during 1999 and 2009 were retrieved from PubMed (www.ncbi.nlm.nih.gov/pubmed). Only the reports in which all hMPV subtypes were screened simultaneously were enrolled to avoid sampling bias (see Table S2 for details). Ten-year epidemic curves of hMPV subtypes and hMPV_A2 sublineages are summarized in Figure 1. The epidemic peak of hMPV_A1 occurred in 2002, whereas hMPV_B1 and _B2 peaked similarly in 2004. As previously reported, the prevalence of all hMPV subtypes except hMPV_A2 fluctuated every two or three years; the prevalence of hMPV_A2 was atypical as it was long-lasting between 2002 and 2009 with a drop in 2007. This atypical prevalence of hMPV_A2 can be attributed mainly to hMPV_A2b, although hMPV_A2a spiked in 2006 (Figure 1).

### Phylogenetic relationships of hMPV subtypes

**Figure 2:**
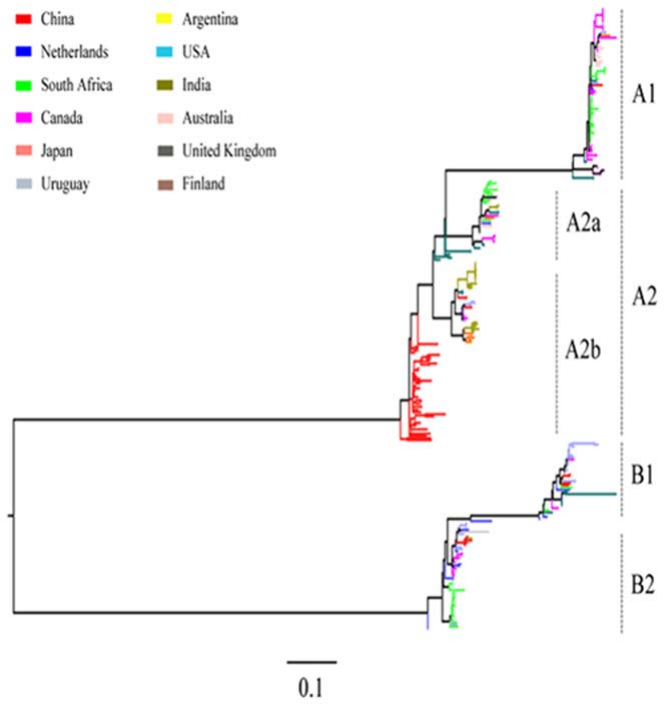
Maximum-likelihood phylogeny of hMPV G genes. A total of 294 partial G gene sequences of hMPV subtypes and sublineages with sampling dates between 1999 and 2009 and of known origin were enrolled. The maximum-likelihood phylogeny was constructed by PAUP* (version 4b10 beta), using a heuristic of Nearest-Neighbor-Interchange (NNI) with 100 bootstrap replicates. The tree is midpoint rooted with a scale bar of 0.1, signifying the genetic distance of nucleotide substitutions per site. For clarity, the background information of each sequence is listed in Table S1.

To unveil the factors that were responsible for the atypical prevalence pattern of hMPV_A2, we first analyzed the phylogenetic relationship of each selected hMPV G sequence to confirm its subtypes. A total of 294 partial G genes corresponding to nucleotide 6431–6760 qualified (GQ153651); all 294 were derived from samples collected between 1981 and 2009 and were subjected to Maximum-likelihood (ML) phylogenetic reconstructions. The data shows well-supported groups of all subtypes and hMPV_A2 sublineages (Figure 2). Phylogeographic analysis revealed a cosmopolitan distribution for the hMPV subtypes and hMPV_A2 sublineages, and strains from a certain geographical location gathered within the same cluster. Co-circulation of multiple subtypes and/or sublineages was observed in some countries, including China, the Netherlands, South Africa, Canada, USA and Australia. To verify these findings using the ML method, the data were analyzed independently using the Neighbor Joining (NJ) and Parsimony methods. Similar tree topologies to that by ML method were obtained (data not shown). The phylogenetic relationship of the hMPV G gene sequences confirms the subtype division of each selected sequence and provides a basis for further analysis of evolutionary dynamics.

### Evolutionary characteristics of hMPV subtypes

To determine why the atypical pattern of hMPV_A2 prevalence occurred, we characterized the evolutionary behavior of the hMPV subtypes. For this purpose, a Bayesian relaxed molecular clock method was used to estimate the evolutionary characteristics of hMPV subtypes. The method assumes no *a priori* correlation between the evolution rate of a lineage and that of its ancestor. The evolutionary rates of the G gene were estimated to be 4.42, 7.81, 8.54, and 4.98×10^−3^ substitutions/site/year for hMPV_A1, A2, B1 and B2, respectively (Table 1). The evolutionary rates of hMPV_A2 and _B1 were almost two-fold those of hMPV_A1 and _B2. Estimation of the genetic distance of each hMPV subtype revealed that hMPV_A2 had the highest overall mean distance of 0.112 (Table 1). These evolutionary characteristics of the hMPV subtypes suggest that hMPV_A2 evolved toward heterogeneity faster than the other subtypes.

**Table 1:** Evolutionary characteristics of hMPV subtypes based on G genes.

hMPV sublineages	Substitution rate (CR^a^)	Overall mean distance (S. E.^b^)
A1	4.42 (2.20–6.78)	0.040 (0.005)
A2	7.81 (6.20–9.52)	0.112 (0.011)
-A2a	7.36 (4.24–10.59)	0.046 (0.006)
-A2b	7.62 (5.79–9.46)	0.092 (0.010)
B1	8.54 (3.98–13.36)	0.049 (0.006)
B2	4.98 (3.53–6.48)	0.056 (0.005)
All	7.40 (5.72–9.15)	0.459 (0.040)

a Substitution rates are expressed as 10^−3^ substitutions per site per year.

b Standard error, derived from 1,000 bootstraps of overall mean distance.

### Dynamics of population growth

**Figure 3:**
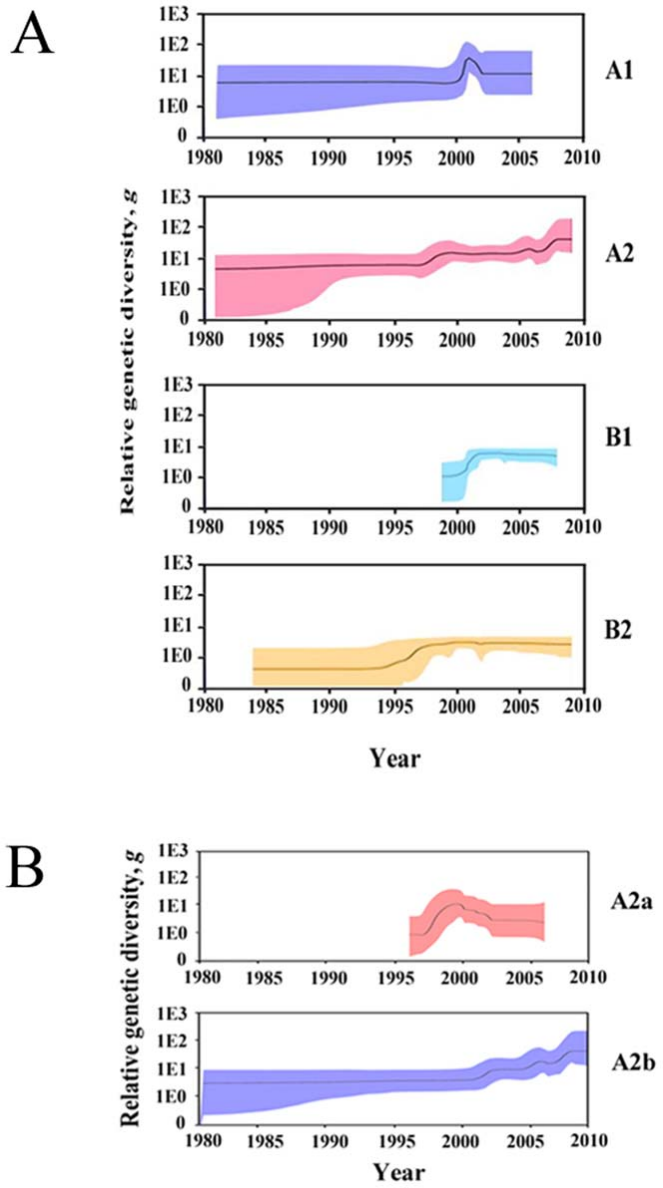
Genetic diversity dynamics of hMPV subtypes and hMPV_A2 sublineages. Bayesian skyline plot estimates depicting the past genetic diversity dynamics of hMPV subtypes (A) and hMPV_A2 sublineages (B) were generated using Bayesian Markov chain Monte Carlo analysis implemented in Beast 1.5.4. The *y* axis of the skyline plot represents genetic diversity (g), which is equal to the product of the effective population size (Ne) and the generation length in years (τ). Black lines represent the median estimate. Color regions show 95% HPD limits.

To infer the demographic history of hMPV, Bayesian skyline plots were used to reconstruct the population histories of hMPV subtypes and hMPV_A2 sublineages from our analysis of the genetic diversity in the G genes over time. hMPV_A1 showed a sharp, but transient, increase in relative genetic diversity (*g*) in 2000 followed by an increase in 2001 and then a steep decrease (Figure 3A). This corresponds to the global increase of hMPV_A1 in 2001 and its decrease in 2002 (Figure 1). The genetic diversity of hMPV_A2 increased in 1997, 2005, and 2007 (Figure 3A). The increases in genetic diversity during 2005 and 2007 correspond to global increases in the epidemic of hMPV_A2. In 1995, hMPV_ B2 exhibited a similar increase in genetic diversity. In 2001, the genetic diversity and epidemic of hMPV_B1 increased, similar to the increases seen in hMPV_A2 (Figure 1). As the increase in genetic diversity of hMPV_B2 occurred before 2001 (the identifying year of hMPV), no direct relationship was observed in its epidemic. The history of relative genetic diversity obtained here largely corresponds to the global epidemic data, demonstrating that the relative genetic diversity of G genes generally reflects the population dynamics of the hMPV subtypes.

The analysis of relative genetic diversity level of hMPV subtypes showed that hMPV_A2 was the highest after 2002 with a value higher than 10, while those of other subtypes were lower than 10. Meanwhile, hMPV_A2 exhibited a rising trend while other subtypes remained steady after the only increase in relative genetic diversity. These findings are correlated with the observation that hMPV_A2 has been the predominant subtype since 2002.

The first increase of the hMPV_A2 prevalence occurred through an increase in the relative genetic diversity of hMPV_A2a around 1997 (Figure 3B); the last two increases of the hMPV_A2 prevalence can be attributed to an increase in the relative genetic diversity of hMPV_A2b. Although the relative genetic diversity of hMPV_A2b has continued to rise since 2001, with a value higher than 10, the relative genetic diversity of hMPV_A2a has remained steady since 2001 with a value lower than 10. These findings further support the fact that hMPV_A2b has been the predominant sublineage of hMPV_A2.

### Natural selection and adaptation of G protein

To investigate the extent of selection pressure on the G gene of the hMPV subtypes and hMPV_A2 sublineages, the mean dN/dS values among branches of the hMPV subtypes were estimated using the M2a codon substitution model in the PAML version 4 [31]. Maximum-likelihood analysis showed that the evolution of hMPV was driven mainly by the signal of purifying selection with estimated values of mean dN/dS ranging from 0.49 to 0.94 for all hMPV subtypes except hMPV_A1. For hMPV A1, the mean dN/dS was 1.25, indicating a signal of positive selection (Table 2). Although hMPV_A2 has atypically prevalent for an extended time period, the mean dN/dS value of hMPV_A2 was the second lowest among hMPV subtypes. The stronger negative selection signal of hMPV_A2 is not surprising, as selection pressure on individual sites may be the key determinant in the survival of hMPV_A2.

**Table 2:** Selection analysis of the G genes of hMPV subtypes.

Subtypes	Mean *dN/dS* (CI^a^)	Positive selection site data			
		Position	AA	dN/dS ± S.E.^b^	Entropy
		82	A	7.74±0.86	1.02
		93	Y	7.74±0.86	0.45
		105	H	7.74±0.86	0.67
		106	S	7.74±0.86	0.72
		124	T	7.74±0.87	0.43
		128	P	7.72±0.96	0.67
		143	K	7.74±0.86	0.45
A1	1.25 (1.23–1.27)	154	P	7.74±0.86	0.68
		155	R	7.53±1.42	0.33
		156	T	7.45±1.51	0.61
		158	S	7.74±0.86	0.87
		165	R	7.67±1.10	0.27
		167	T	7.74±0.86	0.63
		170	T	7.73±0.91	0.34
		93	H	3.53±0.16	0.73
		102	P	3.41±0.32	0.84
		105	N	3.46±0.41	0.82
		106	P	3.37±0.29	0.86
		110	V	3.52±0.16	0.64
		129	S	3.51±0.23	0.48
		143	K	2.85±0.11	0.38
		145	N	3.52±0.10	1.09
		146	P	3.53±0.10	1.25
A2	0.73 (0.67–0.79)	151	N	3.39±0.30	0.93
		154	P	2.78±0.13	0.88
		157	A	2.94±0.15	0.95
		158	T	3.32±0.39	0.66
		172	P	3.53±0.16	0.95
		176	V	3.53±0.16	0.94
		177	F	3.53±0.16	1.10
		70	S	4.78±0.65	0.66
		86	N	4.77±0.67	0.63
		100	E	4.75±0.72	0.39
		105	P	4.76±0.69	0.63
B1	0.50 (0.49–0.51)	109	P	4.77±0.66	0.77
		111	G	4.75±0.73	0.49
		114	Y	4.74±0.75	0.46
		116	G	4.78±0.64	0.68
		136	P	4.67±0.90	0.46
		84	D	5.81±0.64	0.60
		85	P	5.82±0.63	0.83
		93	Q	5.82±0.63	0.64
		105	L	5.61±1.12	0.54
		109	L	5.82±0.63	0.86
B2	0.93 (0.92–0.94)	111	D	5.82±0.63	1.10
		114	H	5.60±1.16	0.30
		119	P	5.75±0.84	0.51
		121	P	5.82±0.63	0.76
		125	V	5.58±1.16	0.72
		140	T	5.82±0.63	0.79

a CI, 95% confidence interval,

b S.E. Standard error.

Because individual site mutations may play a role in the atypical prevalence of hMPV_A2, the positive selection sites of the hMPV subtypes were estimated using a similar codon substitution model (Table 2). We identified 14, 16, 9, and 11 positive selection sites for hMPV_A1, A2, B1, and B2, respectively. Similar values of dN/dS for positive selection sites within subtypes were observed. Moreover, sites of hMPV_A1 had the highest dN/dS values and sites of hMPV_A2 had the lowest values among hMPV subtypes. It seems that the largest number of positive selection sites in hMPV_A2 contribute to the atypical prevalence. An additional entropy analysis on amino acids was also taken into account to gain more supports [32], [35]. Entropy analysis [32] identified 1, 3, 0, and 1 positive selection amino acids for hMPV_A1, A2, B1, and B2, respectively, when the threshold of entropy was set to 1 [35]. Our findings show that because the G sequence of hMPV_A2 had more positive selection sites than those of the other subtypes, antigenic drift resulted in the increased hMPV_2 epidemic [36], [37]. Moreover, the positive selection sites of hMPV_A2a and A2b were inferred independently at the codon and amino acid levels. Although hMPV_A2a had a higher mean dN/dS value at the codon level than hMPV_A2b, hMPV_A2b had more sites with higher entropy at the amino acid level than hMPV_A2a (Table 3). hMPV_A2b had 5 positive selection sites at the amino acid level, whereas hMPV_A2a had 1, suggesting increased severity of the hMPV_A2b epidemic [37].

**Table 3:** Selection analysis of the G genes of hMPV_A2 sublineages.

Sublineages	Mean *dN/dS* (CI^a^)	Positive selection site data			
		Position	AA	dN/dS ± S.E.^b^	Entropy
		110	V	5.146±0.822	0.21
		145	N	5.120±0.875	0.92
		146	P	5.146±0.822	0.78
A2a	0.68 (0.58–0.78)	151	N	5.146±0.823	0.82
		162	S	5.128±0.859	0.67
		172	P	5.021±1.048	0.62
		176	V	5.146±0.822	0.66
		177	F	5.047±0.997	0.48
		93	L	3.399±0.302	0.78
		102	P	3.399±0.303	0.92
		105	N	3.399±0.302	0.92
		106	S	3.398±0.306	0.91
		110	V	3.398±0.480	0.33
A2b	0.82 (0.70–0.94)	126	R	3.392±0.328	0.43
		129	S	3.399±0.302	0.57
		145	K	3.399±0.302	1.06
		146	S	3.399±0.302	1.10
		151	R	3.289±0.578	0.72
		166	T	3.385±0.353	0.74

a CI, 95% confidence interval.

b S.E. Standard error.

**Figure 4:**
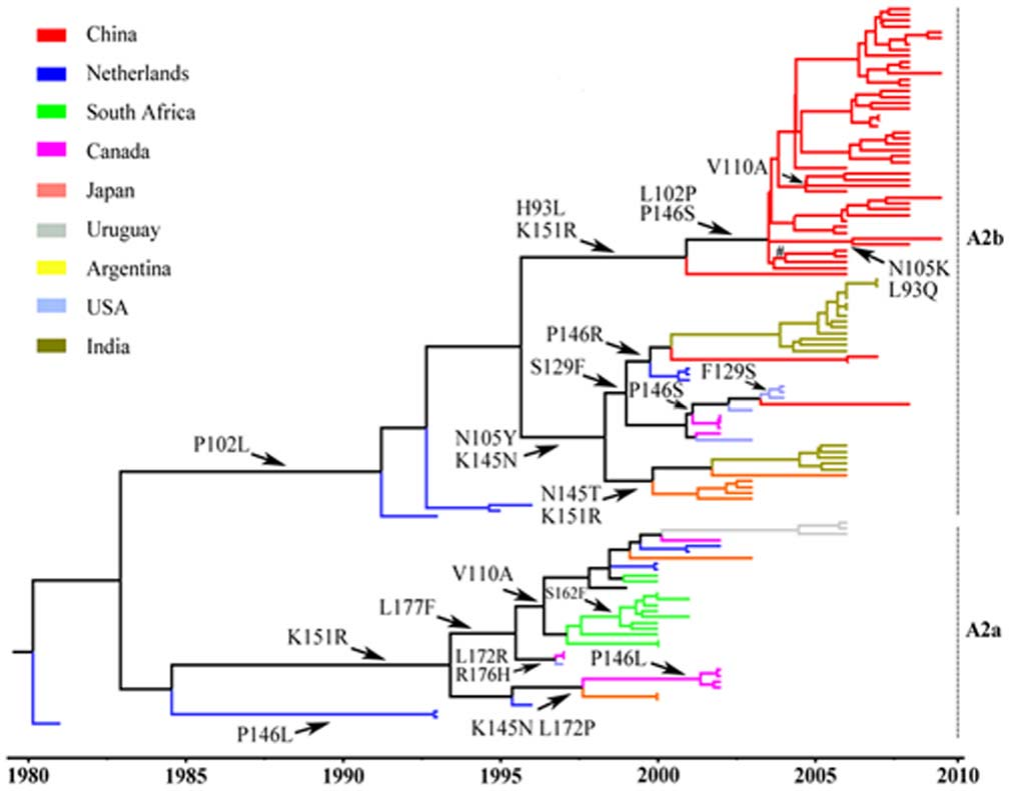
Molecular clock phylogeny of hMPV_A2. The phylogeny was reconstructed using hMPV G sequences longitudinally sampled between 1981 and 2009. Branch lengths are expressed in unit of time.

To trace the positive selection sites in the G gene of hMPV subtypes chronologically, ancestral sequence reconstructions (ASR) were performed with MCC annotated ML trees. The temporal evolution routes of positive selection sites in hMPV_A2a and A2b were drawn on the MCC tree of hMPV_A2 (Figure 4). At least four types of mutation were identified at the positive selection codon sites of hMPV_A2b (Figure 4).There was four reverse changes at positions 93 (H-L-H), 102 (P-L-P), 129 (S-F-S), and 151(K-R-K); a successive change at position 145 (K-N-T between two successive nodes in 1998/2000); two multi-direction changes at positions 105 and 146 (N105K/Y and P146R/S); and two multi-time point changes at positions 146 (P146S in 2002/2003) and 151 (K151R in 2000/2001). However, only two multi-time point changes were observed in hMPV_A2a (P146L in 1992/2001 and L172P in 1995/1997). The mutation hotspots and the diverse mutation types identified for hMPV_A2b suggest that it has been under high selection pressure, consistent with the atypically long-lasting epidemic of hMPV_A2. Mutation hotspots in the temporal evolution routes of other hMPV subtypes were similarly assessed (see Figure S1). Only two multi-time point changes in hMPV_A1 and a successive change in hMPV_B1 were observed. For hMPV_B2, two successive changes and two multi-time point changes were found.

### Phylogeography analysis

**Figure 5:**
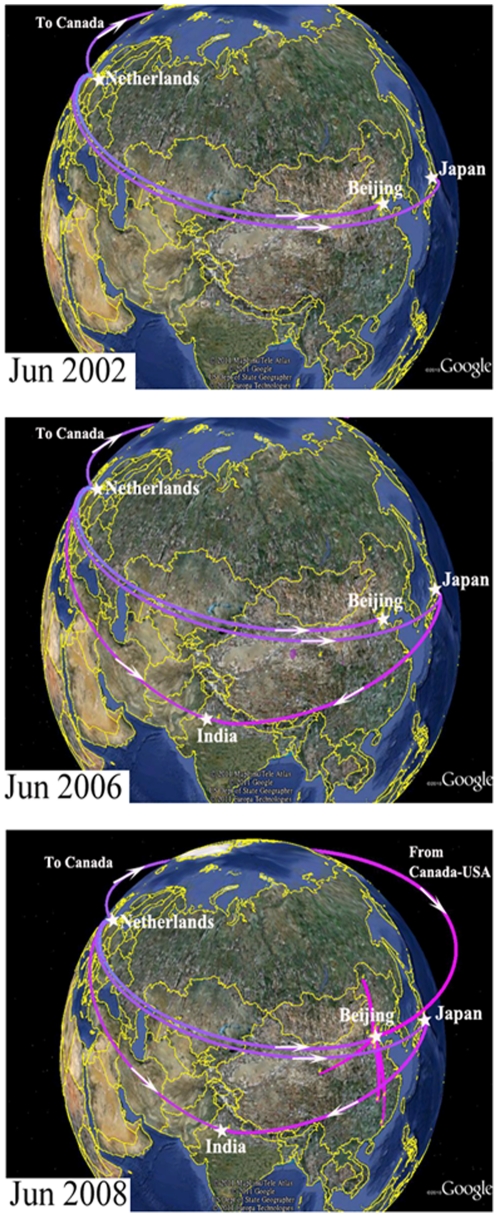
Temporal dynamics of spatial hMPV A2b diffusion. Snapshots of the dispersal pattern for June of 2002, 2006, and 2008 are shown. The location-annotated MCC tree based on the maximum clade credibility tree of hMPV_A2b were converted to KML files for visualizing in Google Earth (http://earth.google.com) (35). Lines between locations represent branches in the MCC tree along which the relevant location transition occurred. The time at the left bottom of each snapshot informs the relative age of the transitions of hMPV_A2b.

**Figure 6:**
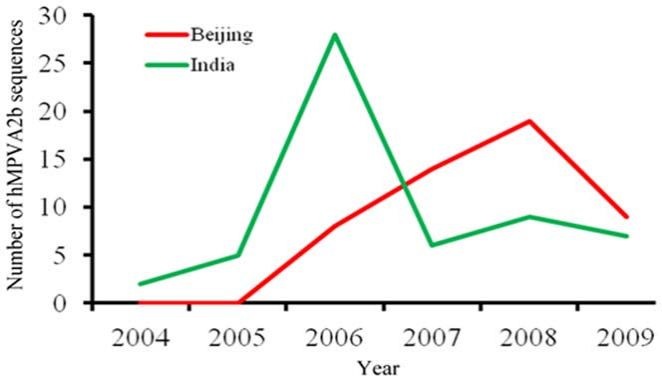
Number of hMPV_A2b sequences deposited to GenBank from India and Beijing, China between 2004 and 2009. G and F gene sequences of hMPV_A2b collected from India and Beijing, China between 2004 and 2009 were retrieved from GenBank. All the G and F sequences were obtained from different isolates to avoid repetitive statistics. Genotyping of these sequences was based on the relative reports and confirmed by Neighbor-joining based phylogeny reconstruction as described previously (55).

To determine the mechanism behind the rise of hMPV_A2b in recent years, the spatial diffusion of the time-scaled genealogy of hMPV_A2b was traced using a recently developed BSSVS approach (Figure 5). By June 2002, hMPV_A2b had emerged in the Netherlands and spread to Canada, Japan, and Beijing, China. By June 2006, the spread routes from the Netherlands and Japan had joined in India, and another route from Canada to USA had emerged. By June 2008, hMPV_A2b had spread from USA to Beijing, China and joined the route from the Netherlands. Coincidently, hMPV_A2b sequences retrieved from GenBank boosted from India in 2006 and Beijing, China in 2008 (Figure 6, Table S3). The consistency between the phylogeographic data and the atypical epidemic of hMPV_A2 show that viral transmission plays an important role in the growth of the virus-infected population, which further explains the atypical epidemic of hMPV_A2b in recent years.

## Discussion

Our analysis of the global prevalence of hMPV subtypes shows a long-lasting epidemic of hMPV_A2 that is atypical in comparison with those of the other hMPV subtypes. To study the mechanism behind this hMPV_A2 prevalence, we assessed phylodynamics, natural selection and adaptation, and phylogeography of the hMPV subtypes and the hMPV_A2 sublineages. Our evolutionary dynamics data are consistent with the atypical epidemic pattern of hMPV_A2 in recent years, providing a molecular evolutionary explanation for the atypical hMPV_A2 epidemic.

The substitution rates of hMPV G genes in this study were estimated to a range of 4.42–8.54×10^−3^ substitutions/site/year, which is similar to those reported by Padhi et al [10], but higher than those of de Graaf et al [38]. We found that hMPV_A2 had the second highest substitution rate after hMPV_B1. However, genetic distance estimation revealed that the overall mean distance of hMPV_A2 is at least two-fold higher than that of all other subtypes. Because hMPV_A2 has the largest overall mean distance and the second-highest substitution rate in G genes, it may have evolved fastest toward heterogeneity, explaining its time serial trend of the effective population size as well as its atypical epidemic.

The Bayesian skyline plots show a general upward trend in the relative genetic diversity of hMPV_A2, which is higher than that of the other hMPV subtypes. This is consistent with its atypical prevalence pattern, as relative genetic diversity level is one determinant of viral survival [39], [40]. Other hMPV subtypes show a steady level of relative genetic diversity after 2005, consistent with their lower prevalence during the same time period. Moreover, most fluctuations in relative genetic diversity presented here largely agree with the global prevalence of all hMPV subtypes, except hMPV_B2, demonstrating that relative genetic diversity can reveal viral features of epidemic dynamics [39]. Insufficient screening of hMPV from clinical specimens might account for the inconsistency between epidemic dynamics and the increased prevalence of hMPV_B2 between 1995 and 1997, as hMPV was not recognized as a human pathogen until 2001. Overall, our data suggest that the pattern of relative genetic diversity matches the corresponding epidemics of hMPV, indicating that the relative dynamics of genetic diversity can be used to explain the patterns of past epidemics of hMPV.

One indication of purifying selection could be seen in mean dN/dS values under 1, which were observed for each hMPV subtype, except hMPV_A1. hMPV_A1 had a mean dN/dS value of 1.25. This strong signal of positive selection in hMPV _A1 seems to reflect an intense fluctuation in its history of genetic diversity. Although hMPV_A2 had the second lowest mean dN/dS value, it had the largest number of positive selection sites. These positive selection sites contained various mutation hotspots in the temporal evolution routes of hMPV_A2b. Fewer positive selection sites were observed in hMPV_A2a than in hMPV_A2b, and fewer mutation hotspots were found in the temporal evolution routes of hMPV_A1, B1, and B2 than in hMPV_A2. These findings show that hMPV_A2 has undergone higher selection pressure in the temporal evolution routes than the other subtypes, consistent with the atypical epidemic of hMPV_A2. Because the epidemic and evolutionary dynamics of hMPV_A2b are similar to that of hMPV_A2, hMPV_A2b may account for the atypical epidemic of hMPV_A2.

We also used phylogeography analysis to investigate the epidemiological history and the diffusion routes of the epidemic hMPV virus. Our Bayesian phylogeography analysis revealed two fusions in the diffusion routes of hMPV_A2b. Because sequences from identical isolates were excluded, our findings suggest that the viral transmission from different regions enhanced the viral capacity against host immunity and thereby intensified the hMPV_A2b epidemic. A possible reason is that host immunity cannot effectively fight against the novel strain of antigenic divergent hMPV_A2b, causing a higher viral prevalence when the novel hMPV_A2b strain was introduced to a specific population. These findings confirm that epidemic history, along with spatial-temporal dynamics of gene flow and phylogeography analysis need to be considered in the planning of prevention strategies, as has previously been suggested [41], [42]

In summary, the evolutionary dynamics of hMPV subtypes determined in this study provide insight into the epidemic and variation of hMPV in recent years. These data can contribute to the diagnosis, surveillance, and control of hMPV as well as for future vaccine development.

## Supporting Information

Figure S1 **Molecular clock phylogenies of hMPV subtypes.** The phylogenies were reconstructed longitudinally using G gene sequences of hMPV subtypes with branch lengths expressed in unit of time.
(TIF)

Table S1 **Sequences information of the hMPV G gene enrolled in this study.**
(DOC)

Table S2 **Global epidemiology of hMPV subtypes during 1999 and 2009.**
(DOC)

Table S3 **G and F sequences of hMPV_A2b submitted to GenBank from India and Beijing, China.**
(DOC)
