# Supplementary figures and images for: Evolutionary Dynamics Analysis of Human Metapneumovirus Subtype A2: Genetic Evidence for Its Dominant Epidemic
Source: PLoS One. 2012 Mar 30;7(3):e34544. doi: 10.1371/journal.pone.0034544 (PMC3316673; doi:10.1371/journal.pone.0034544)

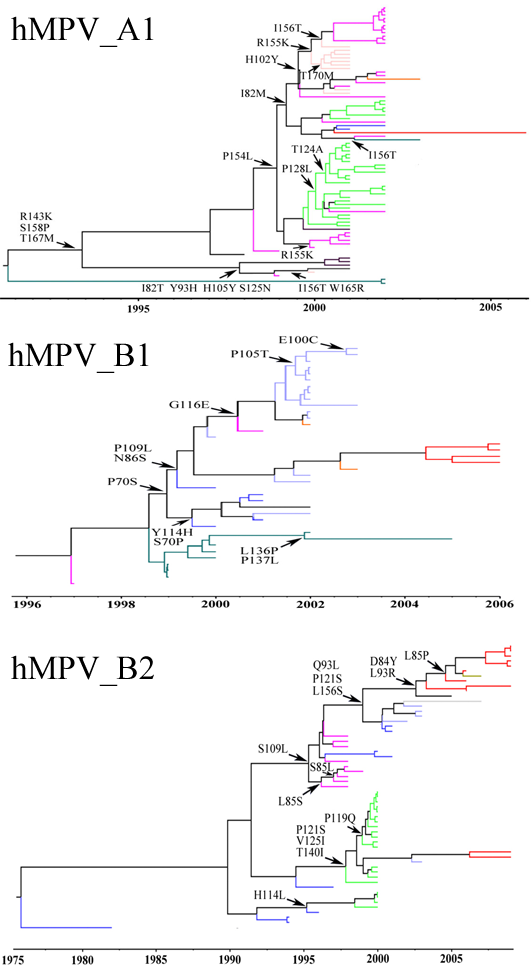

Supplement: Figure S1 — Molecular clock phylogenies of hMPV subtypes. The phylogenies were reconstructed longitudinally using G gene sequences of hMPV subtypes with branch lengths expressed in unit of time. (TIF) [file pone.0034544.s001.tif]
