# Supplementary material for: Evolutionary Dynamics Analysis of Human Metapneumovirus Subtype A2: Genetic Evidence for Its Dominant Epidemic
Source: PLoS One. 2012 Mar 30;7(3):e34544. doi: 10.1371/journal.pone.0034544 (PMC3316673; doi:10.1371/journal.pone.0034544)
Supplement: Table S1 — Sequences information of the hMPV G gene enrolled in this study. (DOC) [file pone.0034544.s002.doc]

**Table S1. Sequences information of the hMPV G gene enrolled in this study**

| GenBank Acc.No. | Country of Isolation | Year of Isolation | Subtype |
| --- | --- | --- | --- |
| DQ362948 | Argentina | 1998 | A1 |
| DQ362950 | Argentina | 2002 | A1 |
| DQ362951 | Argentina | 2002 | A1 |
| DQ362949 | Argentina | 2003 | A1 |
| AF371337 | Australia | 2000 | A1 |
| AY327802 | Australia | 2001 | A1 |
| AY327803 | Australia | 2001 | A1 |
| AY327804 | Australia | 2001 | A1 |
| AY327805 | Australia | 2001 | A1 |
| AY327806 | Australia | 2001 | A1 |
| AY327807 | Australia | 2001 | A1 |
| AY327808 | Australia | 2001 | A1 |
| AY327809 | Australia | 2001 | A1 |
| AY327810 | Australia | 2001 | A1 |
| AY485251 | Canada | 1999 | A1 |
| AY574224 | Canada | 1999 | A1 |
| AY485232 | Canada | 2000 | A1 |
| AY485236 | Canada | 2001 | A1 |
| AY485238 | Canada | 2001 | A1 |
| AY485239 | Canada | 2001 | A1 |
| AY485240 | Canada | 2001 | A1 |
| AY485241 | Canada | 2001 | A1 |
| AY485235 | Canada | 2002 | A1 |
| AY485237 | Canada | 2002 | A1 |
| AY485254 | Canada | 2002 | A1 |
| AY485255 | Canada | 2002 | A1 |
| AY485256 | Canada | 2002 | A1 |
| AY574225 | Canada | 2002 | A1 |
| AY574226 | Canada | 2002 | A1 |
| AY574228 | Canada | 2002 | A1 |
| AY574231 | Canada | 2002 | A1 |
| AY574232 | Canada | 2002 | A1 |
| AY574233 | Canada | 2002 | A1 |
| AY574237 | Canada | 2002 | A1 |
| AY574238 | Canada | 2002 | A1 |
| AY574242 | Canada | 2002 | A1 |
| AY574243 | Canada | 2002 | A1 |
| AY574244 | Canada | 2002 | A1 |
| AY574245 | Canada | 2002 | A1 |
| AY574246 | Canada | 2002 | A1 |
| EF571502 | China | 2006 | A1 |
| AY296015 | Finland | 2001 | A1 |
| AY296016 | Finland | 2001 | A1 |
| AY296017 | Finland | 2001 | A1 |
| AY296018 | Finland | 2001 | A1 |
| AY530092 | Japan | 2003 | A1 |
| AY296019 | the Netherlands | 2001 | A1 |
| AY296020 | the Netherlands | 2002 | A1 |
| AY848881 | Republic of South Africa | 2001 | A1 |
| AY848883 | Republic of South Africa | 2001 | A1 |
| AY848888 | Republic of South Africa | 2001 | A1 |
| AY848889 | Republic of South Africa | 2001 | A1 |
| AY848890 | Republic of South Africa | 2001 | A1 |
| AY848891 | Republic of South Africa | 2001 | A1 |
| AY848892 | Republic of South Africa | 2001 | A1 |
| AY848895 | Republic of South Africa | 2001 | A1 |
| AY848900 | Republic of South Africa | 2001 | A1 |
| AY848902 | Republic of South Africa | 2001 | A1 |
| AY848903 | Republic of South Africa | 2001 | A1 |
| AY848905 | Republic of South Africa | 2001 | A1 |
| AY848906 | Republic of South Africa | 2001 | A1 |
| AY848907 | Republic of South Africa | 2001 | A1 |
| AY848908 | Republic of South Africa | 2001 | A1 |
| AY848909 | Republic of South Africa | 2001 | A1 |
| AY848882 | Republic of South Africa | 2002 | A1 |
| AY848885 | Republic of South Africa | 2002 | A1 |
| AY848886 | Republic of South Africa | 2002 | A1 |
| AY848887 | Republic of South Africa | 2002 | A1 |
| AY848893 | Republic of South Africa | 2002 | A1 |
| AY848894 | Republic of South Africa | 2002 | A1 |
| AY848896 | Republic of South Africa | 2002 | A1 |
| AY848898 | Republic of South Africa | 2002 | A1 |
| AY848899 | Republic of South Africa | 2002 | A1 |
| AY848901 | Republic of South Africa | 2002 | A1 |
| AY848904 | Republic of South Africa | 2002 | A1 |
| AY296014 | United Kingdom | 2001 | A1 |
| DQ362952 | Argentina | 2000 | A2a |
| DQ362953 | Argentina | 2000 | A2a |
| AY297749 | Canada | 1997 | A2a |
| AY485253 | Canada | 1997 | A2a |
| AY485250 | Canada | 1999 | A2a |
| AY485234 | Canada | 2000 | A2a |
| AY574229 | Canada | 2002 | A2a |
| AY574230 | Canada | 2002 | A2a |
| AY574235 | Canada | 2002 | A2a |
| AY574236 | Canada | 2002 | A2a |
| AY574239 | Canada | 2002 | A2a |
| AB530857 | Japan | 2003 | A2a |
| AY296027 | the Netherlands | 1996 | A2a |
| AY296021 | the Netherlands | 2000 | A2a |
| FJ168779 | the Netherlands | 2000 | A2a |
| AY296032 | the Netherlands | 2001 | A2a |
| AY296033 | the Netherlands | 2002 | A2a |
| AY848910 | Republic of South Africa | 2000 | A2a |
| AY848911 | Republic of South Africa | 2000 | A2a |
| AY848912 | Republic of South Africa | 2000 | A2a |
| AY848913 | Republic of South Africa | 2000 | A2a |
| AY848915 | Republic of South Africa | 2000 | A2a |
| AY848917 | Republic of South Africa | 2000 | A2a |
| AY848918 | Republic of South Africa | 2000 | A2a |
| AY848919 | Republic of South Africa | 2000 | A2a |
| AY848914 | Republic of South Africa | 2001 | A2a |
| AY848916 | Republic of South Africa | 2001 | A2a |
| GQ888740 | Uruguay | 2006 | A2a |
| GQ888741 | Uruguay | 2006 | A2a |
| GQ888742 | Uruguay | 2006 | A2a |
| NC004148 | USA | 1997 | A2a |
| AY574227 | Canada | 2002 | A2b |
| AY574234 | Canada | 2002 | A2b |
| AY574240 | Canada | 2002 | A2b |
| AY574241 | Canada | 2002 | A2b |
| EF571504 | China | 2006 | A2b |
| EF571505 | China | 2006 | A2b |
| EF571506 | China | 2006 | A2b |
| EF571508 | China | 2006 | A2b |
| **JN167014*** | **China** | **2006** | **A2b** |
| **JN167015** | **China** | **2006** | **A2b** |
| **JN167016** | **China** | **2006** | **A2b** |
| **JN167017** | **China** | **2006** | **A2b** |
| **JN167018** | **China** | **2006** | **A2b** |
| **JN167020** | **China** | **2006** | **A2b** |
| **JN167021** | **China** | **2006** | **A2b** |
| **JN166985** | **China** | **2007** | **A2b** |
| **JN166986** | **China** | **2007** | **A2b** |
| **JN167019** | **China** | **2007** | **A2b** |
| **JN167022** | **China** | **2007** | **A2b** |
| **JN166987** | **China** | **2008** | **A2b** |
| **JN166988** | **China** | **2008** | **A2b** |
| **JN166989** | **China** | **2008** | **A2b** |
| **JN166990** | **China** | **2008** | **A2b** |
| **JN166991** | **China** | **2008** | **A2b** |
| **JN166996** | **China** | **2008** | **A2b** |
| **JN166997** | **China** | **2008** | **A2b** |
| **JN166998** | **China** | **2008** | **A2b** |
| **JN166999** | **China** | **2008** | **A2b** |
| **JN167000** | **China** | **2008** | **A2b** |
| **JN167001** | **China** | **2008** | **A2b** |
| **JN167002** | **China** | **2008** | **A2b** |
| **JN167003** | **China** | **2008** | **A2b** |
| **JN167004** | **China** | **2008** | **A2b** |
| **JN167005** | **China** | **2008** | **A2b** |
| **JN167006** | **China** | **2008** | **A2b** |
| **JN167007** | **China** | **2008** | **A2b** |
| **JN167008** | **China** | **2008** | **A2b** |
| **JN167009** | **China** | **2008** | **A2b** |
| **JN167010** | **China** | **2008** | **A2b** |
| **JN167011** | **China** | **2008** | **A2b** |
| **JN167012** | **China** | **2008** | **A2b** |
| **JN167013** | **China** | **2008** | **A2b** |
| **JN167023** | **China** | **2008** | **A2b** |
| **JN167024** | **China** | **2008** | **A2b** |
| **JN167025** | **China** | **2008** | **A2b** |
| **JN167026** | **China** | **2008** | **A2b** |
| **JN167027** | **China** | **2008** | **A2b** |
| **JN166992** | **China** | **2009** | **A2b** |
| **JN166993** | **China** | **2009** | **A2b** |
| **JN166994** | **China** | **2009** | **A2b** |
| **JN166995** | **China** | **2009** | **A2b** |
| **JN167028** | **China** | **2009** | **A2b** |
| EU259859 | India | 2006 | A2b |
| EU259860 | India | 2006 | A2b |
| EU259861 | India | 2006 | A2b |
| EU259862 | India | 2006 | A2b |
| EU259863 | India | 2006 | A2b |
| EU259865 | India | 2006 | A2b |
| EU259867 | India | 2006 | A2b |
| EU259868 | India | 2006 | A2b |
| EU259869 | India | 2006 | A2b |
| EU259870 | India | 2006 | A2b |
| EU259871 | India | 2006 | A2b |
| EU259872 | India | 2006 | A2b |
| EU259873 | India | 2006 | A2b |
| EU259874 | India | 2006 | A2b |
| EU259875 | India | 2006 | A2b |
| EU259876 | India | 2006 | A2b |
| EU259864 | India | 2007 | A2b |
| EU259866 | India | 2007 | A2b |
| AY530090 | Japan | 2003 | A2b |
| AY530091 | Japan | 2003 | A2b |
| AY530093 | Japan | 2003 | A2b |
| AY530095 | Japan | 2003 | A2b |
| EF589611 | Japan | 2006 | A2b |
| AY296023 | the Netherlands | 1993 | A2b |
| AY296025 | the Netherlands | 1993 | A2b |
| AY296026 | the Netherlands | 1995 | A2b |
| AY296028 | the Netherlands | 1996 | A2b |
| AY296022 | the Netherlands | 1999 | A2b |
| AY296029 | the Netherlands | 2001 | A2b |
| AY296030 | the Netherlands | 2001 | A2b |
| AY296031 | the Netherlands | 2001 | A2b |
| DQ362955 | Argentina | 1999 | B1 |
| DQ362954 | Argentina | 2000 | B1 |
| DQ362957 | Argentina | 2000 | B1 |
| DQ362958 | Argentina | 2000 | B1 |
| DQ362956 | Argentina | 2002 | B1 |
| GQ888739 | Argentina | 2005 | B1 |
| AY485252 | Canada | 1997 | B1 |
| AY485242 | Canada | 2001 | B1 |
| EF571501 | China | 2006 | B1 |
| EF571503 | China | 2006 | B1 |
| EF571507 | China | 2006 | B1 |
| EF571511 | China | 2006 | B1 |
| **JN167029** | **China** | **2008** | **B1** |
| **JN167030** | **China** | **2008** | **B1** |
| **JN167031** | **China** | **2008** | **B1** |
| **JN167032** | **China** | **2008** | **B1** |
| **JN167033** | **China** | **2008** | **B1** |
| **JN167034** | **China** | **2008** | **B1** |
| **JN167035** | **China** | **2008** | **B1** |
| **JN167036** | **China** | **2008** | **B1** |
| AY530089 | Japan | 2002 | B1 |
| AY530094 | Japan | 2003 | B1 |
| AY296034 | the Netherlands | 1999 | B1 |
| AY525843 | the Netherlands | 1999 | B1 |
| AY296035 | the Netherlands | 2000 | B1 |
| AY296036 | the Netherlands | 2000 | B1 |
| AY296037 | the Netherlands | 2001 | B1 |
| AY296038 | the Netherlands | 2001 | B1 |
| AY296039 | the Netherlands | 2001 | B1 |
| AY848859 | Republic of South Africa | 2002 | B1 |
| AY848860 | Republic of South Africa | 2002 | B1 |
| GQ888744 | Uruguay | 2006 | B1 |
| DQ393716 | USA | 2000 | B1 |
| DQ312445 | USA | 2002 | B1 |
| DQ312446 | USA | 2002 | B1 |
| DQ312447 | USA | 2002 | B1 |
| DQ312448 | USA | 2002 | B1 |
| DQ312450 | USA | 2002 | B1 |
| DQ312452 | USA | 2002 | B1 |
| DQ312454 | USA | 2002 | B1 |
| DQ312455 | USA | 2002 | B1 |
| DQ312459 | USA | 2002 | B1 |
| ADQ312461 | USA | 2002 | B1 |
| DQ393715 | USA | 2002 | B1 |
| DQ393717 | USA | 2003 | B1 |
| DQ393718 | USA | 2003 | B1 |
| DQ393719 | USA | 2003 | B1 |
| AY297748 | Canada | 1998 | B2 |
| AY485243 | Canada | 1998 | B2 |
| AY485245 | Canada | 1998 | B2 |
| AY485246 | Canada | 1998 | B2 |
| AY485247 | Canada | 1998 | B2 |
| AY485248 | Canada | 1998 | B2 |
| AY485249 | Canada | 1998 | B2 |
| AY574247 | Canada | 1999 | B2 |
| EF571500 | China | 2006 | B2 |
| EF571509 | China | 2006 | B2 |
| EF571510 | China | 2006 | B2 |
| **JN167037** | **China** | **2009** | **B2** |
| **JN167038** | **China** | **2009** | **B2** |
| **JN167039** | **China** | **2009** | **B2** |
| **JN167040** | **China** | **2009** | **B2** |
| **JN167041** | **China** | **2009** | **B2** |
| **JN167042** | **China** | **2009** | **B2** |
| **JN167043** | **China** | **2009** | **B2** |
| **JN167044** | **China** | **2009** | **B2** |
| EU259877 | India | 2007 | B2 |
| AY296040 | the Netherlands | 1994 | B2 |
| FJ168778 | the Netherlands | 1994 | B2 |
| AY296042 | the Netherlands | 1996 | B2 |
| AY296043 | the Netherlands | 1997 | B2 |
| AY296041 | the Netherlands | 1999 | B2 |
| AY296044 | the Netherlands | 2000 | B2 |
| AY296045 | the Netherlands | 2001 | B2 |
| AY296046 | the Netherlands | 2001 | B2 |
| AY296047 | the Netherlands | 2001 | B2 |
| AY848861 | Republic of South Africa | 2000 | B2 |
| AY848862 | Republic of South Africa | 2000 | B2 |
| AY848863 | Republic of South Africa | 2000 | B2 |
| AY848864 | Republic of South Africa | 2000 | B2 |
| AY848865 | Republic of South Africa | 2000 | B2 |
| AY848866 | Republic of South Africa | 2000 | B2 |
| AY848867 | Republic of South Africa | 2000 | B2 |
| AY848868 | Republic of South Africa | 2000 | B2 |
| AY848869 | Republic of South Africa | 2000 | B2 |
| AY848870 | Republic of South Africa | 2000 | B2 |
| AY848871 | Republic of South Africa | 2000 | B2 |
| AY848872 | Republic of South Africa | 2000 | B2 |
| AY848873 | Republic of South Africa | 2000 | B2 |
| AY848874 | Republic of South Africa | 2000 | B2 |
| AY848875 | Republic of South Africa | 2000 | B2 |
| AY848876 | Republic of South Africa | 2000 | B2 |
| AY848877 | Republic of South Africa | 2000 | B2 |
| AY848878 | Republic of South Africa | 2000 | B2 |
| AY848879 | Republic of South Africa | 2000 | B2 |
| AY848880 | Republic of South Africa | 2000 | B2 |
| EF535506 | Taiwan | 2000 | B2 |
| GQ888743 | Uruguay | 2007 | B2 |
| DQ312443 | USA | 2002 | B2 |
| DQ312453 | USA | 2003 | B2 |
| DQ312457 | USA | 2003 | B2 |
| DQ312460 | USA | 2003 | B2 |
| DQ393720 | USA | 2003 | B2 |
| DQ312465 | USA | 2004 | B2 |

*Sequences in bold are from this study.
