# Supplementary material for: Evolutionary Dynamics Analysis of Human Metapneumovirus Subtype A2: Genetic Evidence for Its Dominant Epidemic
Source: PLoS One. 2012 Mar 30;7(3):e34544. doi: 10.1371/journal.pone.0034544 (PMC3316673; doi:10.1371/journal.pone.0034544)
Supplement: Table S2 — Global epidemiology of hMPV subtypes during 1999 and 2009. (DOC) [file pone.0034544.s003.doc]

**Table S2**. Global epidemiology of hMPV subtypes during 1999 and 2009

| **Year** | **hMPV subtypes & hMPV_A2 sublineages** | | | | | | | **Reference** ^a^ |
| --- | --- | --- | --- | --- | --- | --- | --- | --- |
|  | **A1** | **A2** | **A2a** | **A2b** | **B1** | **B2** | **Total** |  |
| 1999 | 1 | 0 | 0 | 0 | 0 | 1 | 2 | [1] |
| 2000 | 0 | 8 | 8 | 0 | 0 | 20 | 28 | [1,2] |
| 2001 | 28 | 7 | 4 | 3 | 0 | 0 | 35 | [1-6] |
| 2002 | 82 | 15 | 7 | 8 | 29 | 28 | 154 | [2-10] |
| 2003 | 40 | 47 | 8 | 39 | 23 | 12 | 122 | [3,5-18] |
| 2004 | 33 | 105 | 25 | 80 | 201 | 88 | 427 | [3,5,7-9,11-17,19-24] |
| 2005 | 14 | 116 | 32 | 84 | 35 | 33 | 198 | [11,19-30] |
| 2006 | 3 | 142 | 64 | 78 | 50 | 31 | 226 | [11,13,25-29,31-36] |
| 2007 | 1 | 22 | 7 | 15 | 10 | 17 | 50 | [13,25,26,32,33,36] |
| 2008 | 6 | 57 | 1 | 56 | 10 | 12 | 85 | [33,34] |
| 2009 | 0 | 51 | 0 | 51 | 13 | 14 | 78 | [34-37] |
| Total | 208 | 570 | 156 | 414 | 371 | 256 | 1405 |  |

^a^ References cited in this table are listed in the Supplemented Materials.

**References cited within Table S2**

1. Williams JV, Wang CK, Yang CF, Tollefson SJ, House FS, et al (2006) The role of human metapneumovirus in upper respiratory tract infections in children: a 20-year experience. J Infect Dis 193: 387-395.
2. Ludewick HP, Abed Y, van Niekerk N, Boivin G, Klugman KP, et al (2005) Human metapneumovirus genetic variability, South Africa. Emerg Infect Dis 11: 1074-1078.
3. Sloots TP, Mackay IM, Bialasiewicz S, Jacob KC, McQueen E, et al (2006) Human metapneumovirus, Australia, 2001-2004. Emerg Infect Dis 12: 1263-1266.
4. Bastien N, Ward D, Van Caeseele P, Brandt K, Lee SH, et al (2003) Human metapneumovirus infection in the Canadian population. J Clin Microbiol 41: 4642-4646.
5. Garcia-Garcia ML, Calvo C, Perez-Brena P, De Cea JM, Acosta B, et al (2006) Prevalence and clinical characteristics of human metapneumovirus infections in hospitalized infants in Spain. Pediatr Pulmonol 41: 863-871.
6. Williams JV, Edwards KM, Weinberg GA, Griffin MR, Hall CB, et al (2010) Population-based incidence of human metapneumovirus infection among hospitalized children. J Infect Dis 201: 1890-1898.
7. Mackay IM, Bialasiewicz S, Jacob KC, McQueen E, Arden KE, et al (2006) Genetic diversity of human metapneumovirus over 4 consecutive years in Australia. J Infect Dis 193: 1630-1633.
8. Gerna G, Campanini G, Rovida F, Sarasini A, Lilleri D, et al (2005) Changing circulation rate of human metapneumovirus strains and types among hospitalized pediatric patients during three consecutive winter-spring seasons. Brief report. Arch Virol 150: 2365-2375.
9. Gray GC, Capuano AW, Setterquist SF, Erdman DD, Nobbs ND, et al (2006) Multi-year study of human metapneumovirus infection at a large US Midwestern Medical Referral Center. J Clin Virol 37: 269-276.
10. Gray GC, Capuano AW, Setterquist SF, Sanchez JL, Neville JS, et al (2006) Human metapneumovirus, Peru. Emerg Infect Dis 12: 347-350.
11. Oliveira DB, Durigon EL, Carvalho AC, Leal AL, Souza TS, et al (2009) Epidemiology and genetic variability of human metapneumovirus during a 4-year-long study in Southeastern Brazil. J Med Virol 81: 915-921.
12. Sarasini A, Percivalle E, Rovida F, Campanini G, Genini E, et al (2006) Detection and pathogenicity of human metapneumovirus respiratory infection in pediatric Italian patients during a winter--spring season. J Clin Virol 35: 59-68.
13. Carneiro BM, Yokosawa J, Arbiza J, Costa LF, Mirazo S, et al (2009) Detection of all four human metapneumovirus subtypes in nasopharyngeal specimens from children with respiratory disease in Uberlandia, Brazil. J Med Virol 81: 1814-1818.
14. Escobar C, Luchsinger V, de Oliveira DB, Durigon E, Chnaiderman J, et al (2009) Genetic variability of human metapneumovirus isolated from Chilean children, 2003-2004. J Med Virol 81: 340-344.
15. Foulongne V, Guyon G, Rodiere M, Segondy M (2006) Human metapneumovirus infection in young children hospitalized with respiratory tract disease. Pediatr Infect Dis J 25: 354-359.
16. Foulongne V, Guyon G, Rodiere M, Segondy M (2006) Human metapneumovirus infection in young children hospitalized with respiratory tract disease. Pediatr Infect Dis J 25: 354-359.
17. Regev L, Hindiyeh M, Shulman LM, Barak A, Levy V, et al (2006) Characterization of human metapneumovirus infections in Israel. J Clin Microbiol 44: 1484-1489.
18. Robinson JL, Lee BE, Bastien N, Li Y (2005) Seasonality and clinical features of human metapneumovirus infection in children in Northern Alberta. J Med Virol 76: 98-105.
19. Abiko C, Mizuta K, Itagaki T, Katsushima N, Ito S, et al (2007) Outbreak of human metapneumovirus detected by use of the Vero E6 cell line in isolates collected in Yamagata, Japan, in 2004 and 2005. J Clin Microbiol 45: 1912-1919.
20. Banerjee S, Bharaj P, Sullender W, Kabra SK, Broor S (2007) Human metapneumovirus infections among children with acute respiratory infections seen in a large referral hospital in India. J Clin Virol 38: 70-72.
21. Chung JY, Han TH, Kim BE, Kim CK, Kim SW, et al (2006) Human metapneumovirus infection in hospitalized children with acute respiratory disease in Korea. J Korean Med Sci 21: 838-842.
22. Kaida A, Iritani N, Kubo H, Shiomi M, Kohdera U, et al (2006) Seasonal distribution and phylogenetic analysis of human metapneumovirus among children in Osaka City, Japan. J Clin Virol 35: 394-399.
23. Smuts H, Workman L, Zar HJ (2008) Role of human metapneumovirus, human coronavirus NL63 and human bocavirus in infants and young children with acute wheezing. J Med Virol 80: 906-912.
24. Wang HC, Huang SW, Wang SW, Tsai HP, Kiang D, et al (2008) Co-circulating genetically divergent A2 human metapneumovirus strains among children in southern Taiwan. Arch Virol 153: 2207-2213.
25. Chung JY, Han TH, Kim SW, Hwang ES (2008) Genotype variability of human metapneumovirus, South Korea. J Med Virol 80: 902-905.
26. Caracciolo S, Minini C, Colombrita D, Rossi D, Miglietti N, et al (2008) Human metapneumovirus infection in young children hospitalized with acute respiratory tract disease: virologic and clinical features. Pediatr Infect Dis J 27: 406-412.
27. Boivin G, De Serres G, Hamelin ME, Cote S, Argouin M, et al (2007) An outbreak of severe respiratory tract infection due to human metapneumovirus in a long-term care facility. Clin Infect Dis 44: 1152-1158.
28. Larcher C, Pagani E, Rossi P, Amato B, Pescollderungg L, et al (2008) Comparison of human metapneumovirus genotypes from the province of Bolzano in northern Italy with strains from surrounding regions in Italy and Austria. Jpn J Infect Dis 61: 154-156.
29. Ljubin-Sternak S, Santak M, Cepin-Bogovic J, Bace A, Vojnovic G, et al (2008) Detection of genetic lineages of human metapneumovirus in Croatia during the winter season 2005/2006. J Med Virol 80: 1282-1287.
30. Mao HW, Yang XQ, Zhao XD (2008) Characterization of human metapneumoviruses isolated in Chongqing, China. Chin Med J (Engl) 121: 2254-2257.
31. Camps M, Ricart S, Dimova V, Rovira N, Munoz-Almagro C, et al (2008) Prevalence of human metapneumovirus among hospitalized children younger than 1 year in Catalonia, Spain. J Med Virol 80: 1452-1460.
32. Carr MJ, Waters A, Fenwick F, Toms GL, Hall WW, et al (2008) Molecular epidemiology of human metapneumovirus in Ireland. J Med Virol 80: 510-516.
33. Chen X, Zhang ZY, Zhao Y, Liu EM, Zhao XD (2010) Acute lower respiratory tract infections by human metapneumovirus in children in Southwest China: a 2-year study. Pediatr Pulmonol 45: 824-831.
34. Li XY, Chen JY, Kong M, Su X, Yi YP, et al (2009) Prevalence of human metapneumovirus in hospitalized children with respiratory tract infections in Tianjin, China. Arch Virol 154: 1831-1836.
35. Loo LH, Tan BH, Ng LM, Tee NW, Lin RT, et al (2007) Human metapneumovirus in children, Singapore. Emerg Infect Dis 13: 1396-1398.
36. Pizzorno A, Masner M, Medici C, Sarachaga MJ, Rubio I, et al (2010) Molecular detection and genetic variability of human metapneumovirus in Uruguay. J Med Virol 82: 861-865.
37. Toda S, Kimura H, Noda M, Mizuta K, Matsumoto T, et al (2010) Phylogenetic analysis of human metapneumovirus from children with acute respiratory infection in Yamaguchi, Japan, during summer 2009. Jpn J Infect Dis 63: 139-140.
