# Supplementary material for: Evolutionary Dynamics Analysis of Human Metapneumovirus Subtype A2: Genetic Evidence for Its Dominant Epidemic
Source: PLoS One. 2012 Mar 30;7(3):e34544. doi: 10.1371/journal.pone.0034544 (PMC3316673; doi:10.1371/journal.pone.0034544)
Supplement: Table S3 — G and F sequences of hMPV_A2b submitted to GenBank from India and Beijing, China. (DOC) [file pone.0034544.s004.doc]

**Table S3. G and F sequences of hMPV_A2b submitted to GenBank from India and Beijing, China.**

| **Isolates from Beijing, China** | | | **Isolates from India** | | |
| --- | --- | --- | --- | --- | --- |
| **Accession NO.** | **Gene** | **Year** | **Accession NO.** | **Gene** | **Year** |
| FJ641089 | F | 2007 | DQ855629 | F | 2006 |
| FJ641069 | F | 2006 | DQ855637 | F | 2006 |
| FJ641093 | F | 2008 | DQ855630 | F | 2006 |
| FJ641084 | F | 2007 | DQ855635 | F | 2006 |
| FJ641077 | F | 2007 | DQ855633 | F | 2006 |
| FJ641083 | F | 2007 | DQ083340 | F | 2005 |
| FJ641074 | F | 2007 | DQ083339 | F | 2005 |
| FJ641064 | F | 2006 | DQ083335 | F | 2004 |
| FJ641104 | F | 2008 | DQ083336 | F | 2005 |
| FJ641102 | F | 2008 | DQ083337 | F | 2005 |
| FJ641100 | F | 2008 | DQ083334 | F | 2004 |
| FJ641090 | F | 2007 | DQ855634 | F | 2006 |
| FJ641098 | F | 2008 | HQ559223 | F | 2006 |
| FJ641087 | F | 2007 | DQ855636 | F | 2006 |
| FJ641105 | F | 2008 | HQ559221 | F | 2007 |
| FJ641070 | F | 2006 | DQ083338 | F | 2005 |
| FJ641086 | F | 2007 | HQ599225 | F | 2008 |
| FJ641073 | F | 2006 | HQ599226 | F | 2008 |
| FJ641091 | F | 2007 | HQ599227 | F | 2008 |
| FJ641088 | F | 2007 | DQ855631 | F | 2006 |
| FJ641081 | F | 2007 | DQ855632 | F | 2006 |
| FJ641092 | F | 2007 | HQ599224 | F | 2009 |
| FJ641103 | F | 2008 | EU259872 | G | 2006 |
| FJ641099 | F | 2008 | EU259876 | G | 2006 |
| FJ641096 | F | 2008 | EU259875 | G | 2006 |
| FJ641078 | F | 2007 | EU259873 | G | 2006 |
| FJ641108 | F | 2008 | EU259871 | G | 2006 |
| FJ641107 | F | 2008 | EU259866 | G | 2007 |
| FJ641106 | F | 2008 | EU259865 | G | 2006 |
| FJ641097 | F | 2008 | EU259864 | G | 2007 |
| FJ641094 | F | 2008 | EU259863 | G | 2006 |
| FJ641095 | F | 2008 | EU259869 | G | 2006 |
| FJ641101 | F | 2008 | HQ599204 | G | 2006 |
| FJ641085 | F | 2007 | HQ599198 | G | 2006 |
| HQ262565 | F | 2009 | HQ599202 | G | 2007 |
|  |  |  |  |  |  |
| HQ262554 | F | 2009 | EU259861 | G | 2006 |
| HQ262568 | F | 2009 | EU259860 | G | 2006 |
| HQ262552 | F | 2008 | EU259868 | G | 2006 |
| HQ262556 | F | 2009 | HQ599215 | G | 2009 |
| HQ262560 | F | 2009 | HQ599211 | G | 2008 |
| HQ262550 | F | 2008 | HQ599219 | G | 2009 |
| HQ262567 | F | 2009 | HQ599212 | G | 2008 |
| HQ262563 | F | 2009 | HQ599217 | G | 2009 |
| HQ262562 | F | 2009 | HQ599218 | G | 2009 |
| HQ262553 | F | 2008 | HQ599213 | G | 2007 |
| HQ262561 | F | 2009 | HQ599216 | G | 2009 |
| EF571508 | G | 2006 | HQ599214 | G | 2007 |
| EF571504 | G | 2006 | HQ599208 | G | 2008 |
| EF571505 | G | 2006 | HQ599209 | G | 2008 |
| EF571506 | G | 2006 | HQ599210 | G | 2008 |
|  |  |  | EU259867 | G | 2006 |
|  |  |  | EU259870 | G | 2006 |
|  |  |  | EU259862 | G | 2006 |
|  |  |  | EU259859 | G | 2006 |
|  |  |  | EU259874 | G | 2006 |
|  |  |  | HQ599206 | G | 2009 |
|  |  |  | HQ599207 | G | 2008 |
